# Tumour Microenvironment: Roles of the Aryl Hydrocarbon Receptor, O-GlcNAcylation, Acetyl-CoA and Melatonergic Pathway in Regulating Dynamic Metabolic Interactions across Cell Types—Tumour Microenvironment and Metabolism

**DOI:** 10.3390/ijms22010141

**Published:** 2020-12-25

**Authors:** George Anderson

**Affiliations:** Clinical Research Communications (CRC) Scotland & London, Eccleston Square, London SW1V 6UT, UK; anderson.george@rocketmail.com

**Keywords:** cancer, tumour microenvironment, aryl hydrocarbon receptor, immune, melatonin, mitochondria, acetyl-CoA, treatment, racism

## Abstract

This article reviews the dynamic interactions of the tumour microenvironment, highlighting the roles of acetyl-CoA and melatonergic pathway regulation in determining the interactions between oxidative phosphorylation (OXPHOS) and glycolysis across the array of cells forming the tumour microenvironment. Many of the factors associated with tumour progression and immune resistance, such as yin yang (YY)1 and glycogen synthase kinase (GSK)3β, regulate acetyl-CoA and the melatonergic pathway, thereby having significant impacts on the dynamic interactions of the different types of cells present in the tumour microenvironment. The association of the aryl hydrocarbon receptor (AhR) with immune suppression in the tumour microenvironment may be mediated by the AhR-induced cytochrome P450 (CYP)1b1-driven ‘backward’ conversion of melatonin to its immediate precursor N-acetylserotonin (NAS). NAS within tumours and released from tumour microenvironment cells activates the brain-derived neurotrophic factor (BDNF) receptor, TrkB, thereby increasing the survival and proliferation of cancer stem-like cells. Acetyl-CoA is a crucial co-substrate for initiation of the melatonergic pathway, as well as co-ordinating the interactions of OXPHOS and glycolysis in all cells of the tumour microenvironment. This provides a model of the tumour microenvironment that emphasises the roles of acetyl-CoA and the melatonergic pathway in shaping the dynamic intercellular metabolic interactions of the various cells within the tumour microenvironment. The potentiation of YY1 and GSK3β by O-GlcNAcylation will drive changes in metabolism in tumours and tumour microenvironment cells in association with their regulation of the melatonergic pathway. The emphasis on metabolic interactions across cell types in the tumour microenvironment provides novel future research and treatment directions.

## 1. Introduction

Recent work on the pathophysiology of the severe acute respiratory syndrome-coronavirus (SARS-CoV)-2 in the COVID-19 pandemic has highlighted the co-ordinating role of the aryl hydrocarbon receptor (AhR), including in the suppression of CD8+ T cell and natural killer (NK) cell cytotoxicity [1]. A dysregulated inflammatory response, coupled to suppression of the antiviral and anticancer responses of CD8+ T cells and NK cells has emerged as a commonality in the altered immune responses evident in SARS-CoV-2 infection and the tumour microenvironment [1]. AhR activation in CD8+ T cells and NK cells is one of a number of processes contributing to ‘exhaustion’ in these cells, and thereby to the immune suppression that is evident in the tumour microenvironment of almost all cancers. Other pathways also induce an ‘exhausted’ phenotype, including cancer cell release of transforming growth factor (TGF)-β, the activation of the adenosine A2A receptor (A2Ar) and the induction of the cyclooxygenase (COX)2-prostaglandin (PG)E2 path leading to the activation of the PGE2 receptor, (EP)4. Antagonists of these specific ‘immune checkpoint’ pathways are widely utilised in the treatment of cancer patients, often adjunctive and with relatively limited clinical efficacy [2]. Other inhibitors of NK cells, include NKG2A, inhibitory killer cell Ig-like receptors (KIRs), and CD96, whereas NKG2D, CD16, NKp30, NKp44, and NKp46 can activate NK cells. The effects of these inhibitory and activating receptors are classically associated with their differential activation by cancer cell-expressing/released ligands [3]. Overall, a number of factors, including cancer cell derived, can regulate cytolytic cell cytotoxicity and the ‘exhaustion’ associated with immune suppression in the tumour environment.

Partly as a consequence of the limitations of treatment directed towards ‘exhaustion’-inducing pathways, recent work has focussed on the targeting of specific receptors associated with ‘exhaustion’ and which may contribute to this phenotype, especially antagonists of the programmed cell death (PD)-1 receptor and its ligand, PD-L1. However, although there was high expectation of anti-PD-1 and anti-PD-L1 therapy, the efficacy of this treatment approach has also been limited, including from the high levels of immune-related adverse events [4,5]. Such approaches have also come under some criticism due to the limitations in the pathways targeted for treatment and the perhaps superficial targeting of plasma membrane receptors, and thereby avoiding the complexities of the metabolic processes that underpin ‘exhaustion’ [6].

The current article reviews the complexities of the metabolic alterations and interactions across the different cells of the tumour microenvironment that underpin the ‘exhausted’ CD8+ T cell and NK cell phenotypes. As with all immune cells, the process of activation requires cytolytic cells to increase glycolysis. Many of the factors contributing to an ‘exhausted’ phenotype seem to act primarily on preventing glycolysis upregulation. However, the necessity of increased glycolysis is not indicative of a need to shift energy production from oxidative phosphorylation (OXPHOS), as some maintenance of OXPHOS is a prerequisite for these cells to become activated. It is proposed that the maintained production and availability of acetyl-CoA, an indicant of OXPHOS, is a determinant of glycolysis upregulation, with acetyl-CoA acting to regulate the many factors and intracellular pathways contributing to ‘exhaustion’, including TGF-β, adenosine A2Ar activation and AhR/COX2/PGE2/EP4 activation, as well as the effects of obesity and type II diabetes on cancer risk, which are partly driven by raised leptin levels priming cytolytic cells [7]. An underexplored consequence of variations in acetyl-CoA is the impact that this has on the regulation of the melatonergic pathway, with acetyl-CoA being a necessary co-substrate for aralkylamine N-acetyltransferase (AANAT), the initial enzyme in the melatonergic pathway. Consequently, the effects of acetyl-CoA in the regulation of OXPHOS and glycolysis may be intimately linked to its regulation of the melatonergic pathway within mitochondria as well as in the cytoplasm. The AhR is an important driver of many aspects of the ‘exhausted’ phenotype pathophysiology, including via regulation of the COX2/PGE2/EP4 pathway, TGF-β and the adenosine A2Ar, as well as the melatonergic pathway. Treatment implications and future research are indicated, including a role for racial discrimination stress in contributing to the increased severity/fatality of a range of cancers, as seen in African-Americans versus European-Americans, via an increase in pro-inflammatory cytokines and indoleamine 2,3-dioxygenase (IDO) induction, leading to kynurenine activation of the AhR.

## 2. Tumour Microenvironment and Immune Suppression: Exhaustion

A number of different cells are evident in the tumour microenvironment, including different types of cancer cells and a range of immune cells, with the interactions of these cells proposed to ultimately drive immune suppression via their regulation of NK cells and CD8+ T cells. Factors associated with the induction of ‘exhaustion’ in CD8+ T cells and NK cells within the tumour microenvironment, include the AhR, TGF-β, CD73 (ecto-5-Nucleotidase), adenosine A2Ar activation, PD-1, and the COX2/PGE2/EP4 pathway. We briefly review the data pertaining to the functioning of these receptors and pathways, and their contribution of an ‘exhausted’ state in cytolytic cells.

### 2.1. Aryl Hydrocarbon Receptor

The AhR has a number of exogenous and endogenous ligands, including air pollutants, 6-formylindolo[3,2-b]carbazole (FICZ) and the cigarette smoke constituent, 2,3,7,8-tetrachlorodibenzo-p-dioxin (TCDD). However, perhaps more important to its regulation of the cytolytic immune response to viruses and cancers is the induced AhR ligand, kynurenine. Stress and pro-inflammatory cytokines, including interleukin (IL)-1β, IL-6, IL-18, tumour necrosis factor (TNF)α, and especially interferon (IFN)γ, increase indoleamine 2,3-dioxygenase (IDO), which takes tryptophan away from serotonin and melatonin synthesis and drives it to the production of kynurenine and kynurenine pathway products. In some cells, tryptophan 2,3-dioxygenase (TDO) is predominantly expressed and likewise converts tryptophan to kynurenine. The cytokine/IDO/kynurenine/AhR pathway is relevant to a wide array of diverse medical conditions, including neuropsychiatric conditions [8,9], as well as all cancers [10].

The cytokine/IDO/kynurenine/AhR pathway is present within cancer cells, with intracellular AhR activation affording protection in cancer cells [11]. As such, an initial increased release of pro-inflammatory cytokines, especially IFNγ, from NK cells in the tumour microenvironment drives IDO induction in cancer cells, leading to intracrine kynurenine activation of the AhR that acts to protect cancer cells. As well as intracrine kynurenine effects, cancer cells also release kynurenine, which activates the AhR on tumour microenvironment cells, including CD8+ T cells and NK cells, thereby inducing an ‘exhausted’ phenotype, concurrent to increasing PD-1 plasma membrane expression [12]. AhR activation also induces the COX2/PGE2/EP4 pathway [13,14], which is an important driver of an ‘exhausted’ phenotype [15]. Consequently, kynurenine activation of the AhR affords protection within cancer cells and acts to induce ‘exhaustion’ in cytolytic cells, with effects involving the upregulation of COX2 and PD-1. AhR activation also induces CD39 and CD73, thereby increasing the conversion of ATP to adenosine [16], allowing AhR activation to raise adenosine levels from different cells of the tumour microenvironment, thereby driving A2Ar activation and contributing to ‘exhaustion’ in NK cells and CD8+ T cells.

#### AhR and Melatonergic Pathway

**Figure 1:**
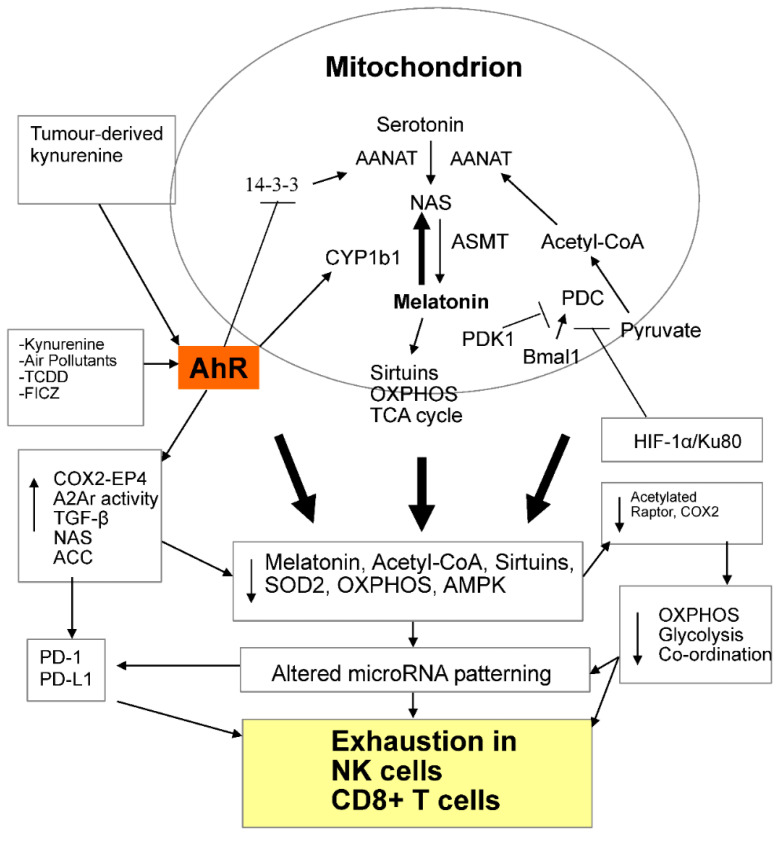
Shows how AhR ligands, such as air pollutants, FICZ, TCDD and kynurenine, including tumour derived, activate the AhR to modulate mitochondrial metabolism, partly via the regulation of the melatonergic pathway. The melatonergic pathway is shown in the mitochondrion, but is also present in the cytoplasm. AhR activation drives a number of processes and factors underpinning exhaustion and tumour survival, including the COX2/PGE2/EP4, adenosine A2Ar, TGF-β, ACC and NAS. In NK cells and CD8+ T cells, factors inhibiting acetyl-CoA, such as HIF-1α/Ku80 and PDK1 inducers, will inhibit the TCA cycle, OXPHOS and the co-ordination of OXPHOS with glycolysis, in association with a decreased acetylation of COX2. OXPHOS and glycolysis are co-ordinated by a decreased acetylation of Raptor within the mTORC1 complex. The changes in mitochondrial function driven by decreased melatonin, acetyl-CoA, Sirtuins, SOD2, OXPHOS and AMPK will modulate gene expression, partly via levels of ROS production and the associated changes in miRNAs expressed. Altered miRNA patterning will drive variations in the classical immune checkpoint inhibitors, such as the PD-1 suppression by miR-138. Variations in such acetyl-CoA, melatonergic pathway regulation of OXPHOS and glycolysis will occur in the array of immune cells within the tumour microenvironment, with the dynamic intracellular and intercellular interactions of these cells driven by variations in metabolic regulation. Other factors may also ‘backward’ convert melatonin to NAS, including activation of the P2Y1 and mGluR5 receptors, as well as O-demethylation, which are not shown for clarity. The processes highlighted in the figure indicate an important AhR role in the regulation of mitochondrial function. However, the temporal and functional specifics of this await determination in the different cells of the tumour microenvironment, including the interactions of AhR, O-GlcNAcylation, acetyl-CoA and melatonergic pathway. The interactions of AhR effects on mitochondrial function with wider AhR effects on transcription in the different cells of the tumour microenvironment will also be important to determine. Arrows indicate increases/induces; T-arrows indicate suppresses. Abbreviations: AANAT: aralkylamine N-acetyltransferase; ACC: acetyl-CoA carboxylase; acetyl-CoA: acetyl-coenzyme A; AhR: aryl hydrocarbon receptor; AMPK: AMP-activated protein kinase; ASMT: acetylserotonin methyltransferase; CD8+: cluster of differentiation 8; COX2: cyclooxygenase 2; CYP: cytochrome P450; EP4: Prostaglandin E2 receptor 4; FICZ: 6-formylindolo[3,2-b]carbazole; HIF: hypoxia-inducible factor; mTORC1: mammalian target of rapamycin complex 1; NK: natural killer; OXPHOS: oxidative phosphorylation; NAS: N-acetylserotonin; PD-1: programmed cell death-1: PD-L1: programmed cell death ligand 1; PDC: pyruvate dehydrogenase complex; PDK: pyruvate dehydrogenase kinase; SOD: superoxide dismutase; TCA: tricarboxylic acid; TCDD: 2,3,7,8-tetrachlorodibenzo-p-dioxin; TGF: transforming growth factor.

The AhR also regulates the melatonergic pathway. The melatonergic pathway is initiated via the conversion of serotonin to N-acetylserotonin (NAS) by AANAT, which is then converted to melatonin by acetylserotonin-methyl-transferase (ASMT). By decreasing tryptophan availability for serotonin synthesis, cytokine-induced IDO/TDO suppresses the serotonin/melatonergic pathway, whilst concurrently increasing the AhR ligands, kynurenine and kynurenic acid. The AhR regulates the melatonergic pathway by a number of means, including via AhR-induced cytochrome P450 (CYP)1B1, which ‘backward’ converts melatonin to NAS. This may be of particular relevance in the tumour microenvironment, given that NAS is a brain-derived neurotrophic factor (BDNF) mimic, via its activation at the BDNF receptor, TrkB [17]. By increasing the NAS/melatonin ratio, AhR-induced NAS activates TrkB, which can enhance the survival and proliferation of cancer stem-like cells (CSC) [18]. Such trophic effects of NAS may arise from any of the cells forming the tumour microenvironment, including NK cells and CD8+ t cells. As 14-3-3ζ/δ (YWHAZ) is crucial for AANAT stabilisation, the AhR inhibition of14-3-3ζ/δ [19], including via microRNAs, allows the AhR to suppress both NAS and melatonin production. As the melatonergic pathway seems to be evident in the cytoplasm and mitochondria of all investigated cells, the AhR may therefore exert some of its effect in different cellular compartments via melatonergic pathway regulation. The antioxidant, immune-regulatory, mitochondria-optimising and cancer apoptosis-inducing effects of melatonin, coupled to NAS trophic effects, make AhR regulation of the melatonergic pathway an important aspect of tumours and the tumour microenvironment [8] as well as chemoresistance [20]. See Figure 1.

The roles of the melatonergic pathway in CD8+ T cells and NK cells have still to be investigated. This is surprising, given the impact of the melatonergic pathway on mitochondrial function, sirtuins (SIRT) and antioxidant enzymes [21,22]. As well as expression within mitochondria, the melatonergic pathway can also be present in the cytoplasm. The expression and activation of the melatonergic pathway in the different cellular compartments of cytolytic cells over the course of activation and ‘exhaustion’ urgently requires investigation. Cytoplasmic 14-3-3ζ is a significant regulator of CD8+ T cell function [23], with effects that include AANAT stabilisation. The AhR regulates 14-3-3ζ and therefore the initiation of the melatonergic pathway [19]. However, the AhR upregulation of the NAS/melatonin ratio, by AhR-induced CYP1B1, would allow any melatonergic pathway activity to increase NAS, which if released would activate TrkB on CSC. Consequently, cancer cell induction of ‘exhaustion’ in NK cells and CD8+ T cells via kynurenine activation of the AhR [18,20] may allow these cells to be a source of trophic support for CSC [20].

Other factors can also lead to the backward conversion of melatonin to NAS, including activation of purinergic (P2Y1), metabotropic glutamate (mGluR5) receptors, as well as O-demethylation [20]. P2Y1, P2Y2, and P2X3 receptor activation by ATP decreases NK cell cytotoxicity [24]. The mGluR5 receptor is expressed in CD8+ T cells [25]. As both extracellular ATP and glutamate are variably increased in the tumour microenvironment, both will modulate cytolytic cell function, partly via the purinergic and glutamatergic regulation of the melatonergic pathway that act to raise the NAS/melatonin ratio. As such, the AhR activation as well as extracellular glutamate and ATP may modulate not only NK cell and CD8+ T cell cytotoxicity, but also cancer cell trophic support, via impacts on the cytosolic and mitochondrial melatonergic pathway.

As acetyl-CoA is a necessary co-substrate for AANAT and the initiation of the melatonergic pathway, the significant role of acetyl-CoA in co-ordinating the mitochondrial OXPHOS status with glycolysis in cytolytic cell activation, as detailed below, would also involve significant modulation of the melatonergic pathway. As such, key factors that act to regulate metabolism and ‘exhaustion’, viz the AhR and acetyl-CoA, may be mediating effects partly via the melatonergic pathway. See Figure 1.

### 2.2. Transforming Growth Factor-β (TGF-β)

TGF-β release, including from tumour cells, can also induce ‘exhaustion’ in CD8+ T cells [26] and NK cells within the tumour microenvironment [27,28]. As well as tumour cells, TGF-β is also released from myeloid-derived suppressor cells (MDSC), tumour-associated M2-like macrophages, gammaDelta (γδ) T cells, mast cells and regulatory T cells (Treg), allowing these cells to contribute to cytolytic cell exhaustion and immune suppression [29,30,31]. Activation of the AhR/COX2/PGE2 pathway in tumours leads to PGE2 release and EP2 and EP4 activation in different cells, in turn increasing TGF-β release, and TGF-β-induced adenosine, from these cells and thereby contributing to cytolytic cell ‘exhaustion’ [32]. As such, the regulation of TGF-β, across cells of the tumour microenvironment is important to the regulation of immune suppression, including following AhR activation. Such AhR effects may then be co-ordinated with decreased melatonin production across cells, leading to the loss of melatonin’s suppression of TGF-β as found not only in cancer cells [33], but also in fibrosis where melatonin’s suppression of TGF-β is a major inhibitor of fibrosis, across different organs and tissues [34,35]. As such, the co-ordinated suppression of melatonin production and raised TGF-β allows AhR activation to heighten TGF-β levels and effects across the various cells of the tumour microenvironment.

Platelets are another important source for TGF-β in the tumour microenvironment, with platelet TGF-β contributing to tumour survival and metastasis [36] as well as the chemoattraction of MDSCs [37]. As AhR activation on platelets increases platelet aggregation and primes platelets for enhanced activation [38], the platelet AhR will be important in the regulation of immune suppression. Melatonin, which is decreased following activation of the cytokine/IDO/AhR path, suppresses platelet activation, suggesting that suppressed melatonin production, both circadian and local, will contribute to processes upregulating platelet TGF-β and its effects [39]. TGF-β can also induce ‘exhaustion’ in cytolytic cells via the upregulation of CD73 and thereby the conversion of adenosine monophosphate (AMP) to adenosine leading to A2Ar activation, as shown in CD4+ T cells [40] and CD8+ T cells [41]. Heightened adenosine production is evident across a number of cells in the tumour microenvironment, highlighting how the dynamic interactions of the array of tumour microenvironment cells can contribute to immune suppression [42].

### 2.3. Hypoxia/HIF-1α Increases A2Ar, CD73 and CD39

Tumour microenvironment hypoxia is a major driver of adenosine production, via the upregulation of hypoxia-inducible factor (HIF)-1α [43]. HIF-1α increases A2Ar, CD73 and CD39, the latter converting ATP to AMP, thereby increasing adenosine and A2Ar activation across a number of cells in the tumour microenvironment [44]. Consequently, eliminating solid tumour hypoxia by natural blood substitutes and synthetic oxygenation agents have been proposed as routes whereby the hypoxia-HIF-1α-adenosine/A2Ar pathway may be blocked, thereby enhancing immunotherapy-targeted treatments [44].

Notably, melatonin significantly inhibits HIF-1α induction [45], being another route, along with antagonism of the AhR and TGF-β, whereby the suppression of melatonin will contribute to immune suppression driven by adenosine A2Ar activation. Hypoxia/HIF1-α alters OXPHOS and mitochondrial metabolism in tumours by inducing Ku80, which binds and activates the pyruvate dehydrogenase kinase (PDK)1 promoter, thereby acting to inhibit the pyruvate dehydrogenase complex (PDC), in turn inhibiting the PDC conversion of pyruvate to acetyl-CoA, which is crucial to driving OXPHOS, the TCA cycle and the melatonergic pathway [46]. See Figure 1. Melatonin degrades and inhibits HIF1-α in tumours, thereby increasing apoptosis [46], again indicating the impact that variations in local melatonin production can have on core tumour regulators [47]. Taking pyruvate away from acetyl-CoA production and driving it to lactate production is an important mediator of key changes in tumour metabolism, as indicated by acetate supplementation leading to tumour cell differentiation under hypoxia [48]. Although, the authors attribute the benefits of acetate supplementation to be mediated by increased chromatin acetylation, it is clear that acetate supplementation would also increase the activation of the melatonergic pathway, TCA cycle and OXPHOS. The relevance of the HIF1-α/Ku80/PDK1 pathway to alterations in metabolism of other cells in the tumour microenvironment will be important to determine.

### 2.4. Adenosine A2Ar, mTORC1, OXPHOS and Exhaustion

A2Ar activation is proposed to mediate cytolytic cell suppression via the cyclic adenosine monophosphate (cAMP)/protein kinase (PK)A/phosphorylated cAMP response element binding protein (pCREB) pathway and mammalian target of rapamycin complex 1 (mTORC1) complex suppression (via S6 phosphorylation), with this leading to impaired T cell receptor (TCR)-dependent extracellular-signal-regulated-kinase (ERK) phosphorylation in human CD8+ T cells [49]. The mTORC1 complex is crucial to glycolysis upregulation in CD8+ T cells and NK cells and is upregulated by a number of processes/factors, including HIF-1α, c-MYC and especially the system L neutral amino acid transporter (LAT)1, encoded by SLC7A5. Upregulated glycolysis is required for all immune cells to become activated, including CD8+ T cells and NK cells [50]. The inhibition of the mTORC1 complex and the prevention of LAT1 upregulation, prevents the necessary induction of glycolysis and therefore the increased metabolism and amino acid availability that is required for a shift to a cytotoxic cellular phenotype. Factors, such as A2Ar activity, which impact on mTORC1 activation and LAT1 upregulation, are therefore crucial in determining whether cytolytic cells shift to a cytotoxic phenotype or state of ‘exhaustion’ [51,52].

Importantly, A2Ar activation not only suppresses glycolysis, but also OXPHOS in CD8+ T cells [49]. This may be of some importance as the suppressive effects of AhR-induced COX2/PGE2/EP4 activation and mTORC1 inhibition in suppressing cytolytic cell glycolysis may be intimately regulated by variations in mitochondrial OXPHOS and acetyl-CoA regulation, allowing acetyl-CoA to co-ordinate OXPHOS and glycolysis. Glycolysis upregulation does not replace OXPHOS in cytolytic cells. Rather, activation of CD8+ T cells and NK cells requires glycolysis upregulation that is coupled to maintained OXPHOS. This indicates interactions of OXPHOS with mTORC1/LAT1-driven glycolysis. Processes relevant to such OXPHOS–glycolysis interactions are seen in other cell types, where cytosolic acetyl-CoA levels are crucial to the acetylation of the mTORC1 complex factor, Raptor. Raptor deacetylation leads to a reduction in the lysosomal localisation of mTOR, thereby impairing mTORC1 activation and function [53]. Decreased acetyl-CoA may therefore link suppressed OXPHOS and melatonergic pathway function with lower mTORC1-driven glycolysis in ‘exhausted’ CD8+ T cells and NK cells. The acetylation of COX2 also inhibits COX2 [54], indicating that acetyl-CoA may directly regulate key processes of glycolysis/cytotoxicity induction (mTORC1) and glycolysis/cytotoxicity suppression (COX2) in cytolytic cells. Consequently, PDK1 inhibition and the associated PDC disinhibition, which drives the conversion of pyruvate to acetyl-CoA, are important regulators of the array of diverse receptors and pathways linked to the induction of ‘exhaustion’ in cytolytic cells. The maintenance of acetyl-CoA levels therefore underpin why maintained OXPHOS is essential to cytotoxic functions of CD8+ T cells and NK cells. See Figure 1.

### 2.5. Acetyl-CoA, AhR and AMPK

A deficiency in AMP-activated protein kinase (AMPK) is long appreciated to contribute to exhaustion in CD8+ T cells [55], including by increasing PD-1 [56]. AMPK also inhibits acetyl-CoA carboxylase (ACC), thereby increasing acetyl-CoA and ATP synthesis [57]. Notably, AMPK is regulated by the AhR-driven degradation of Synphilin-1, thereby decreasing AMPK and therefore preventing the AMPK suppression of ACC [58]. As such, the AhR degradation of Synphilin-1, leading to AMPK inhibition and ACC disinhibition is another route whereby the AhR can attenuate acetyl-CoA levels, thereby lowering acetylated-COX2, acetylated-Raptor and the melatonergic pathways, with consequences for the mTORC1 complex induction of cMYC, LAT1 and glycolysis. See Figure 1. It will be important to determine as to whether the acetylation of COX2 modulates the ability of the AhR to suppress Synphilin-1. Similar processes are evident in NK cells.

AMPK inhibition underpins the association of obesity and type II diabetes with many cancers. High glucose levels inhibit AMPK, coupled to MHC class I chain-related protein A/B (MICA/B) suppression [59], thereby contributing to NK cell ‘exhaustion’ in obesity and type II diabetes. Metformin, commonly used to treat type II diabetes, can upregulate CD8+ T cell cytotoxicity via an increase in AMPK, as well as by suppressing adenosine production in MDSCs, via the inhibition of CD39 and CD73, and therefore A2Ar activity in cytolytic cells [60]. As such, the AMPK interaction with nutrient-sensing, mTOR and metabolism may all be subject to AhR regulation, with important effects mediated by the suppression of acetyl-CoA levels.

Overall, the interactions of the AhR with regulators of PDC-regulated acetyl-CoA provides a metabolic framework that integrates wider bodies of data pertaining to the induction of ‘exhaustion’ in CD8+ T cells and NK cells in the tumour microenvironment, with effects mediated via alterations in the dynamic metabolic interactions of tumour microenvironment cells, with key metabolic processes indicated in Figure 1.

## 3. Integrating Wider Bodies of Data: Ageing, Sirtuins, Circadian and Cell-Specific Factors

Figure 1 provides a metabolic framework for integrating wider bodies of data pertaining to how the intercellular interactions of the tumour microenvironment drive ‘exhaustion’ in CD8+ T cells and NK cells. Alterations in the regulation of mitochondrial metabolism, including the regulation of acetyl-CoA and the melatonergic pathway, are crucial in all cells of the tumour microenvironment.

### 3.1. MDSCs, Dendritic Cells, Metabolism and Acetyl-CoA

As noted, alterations in other cells in the tumour microenvironment can modulate CD8+ T cell and NK cell cytotoxicity, including via variations in fluxes from MDSC, tumour-associated M2-like macrophages, γδT cells, mast cells, Treg, neutrophils and dendritic cells, allowing these cells to contribute to the regulation of cytolytic cell exhaustion and immune suppression [29,30,31,61]. The suppression of tuberous sclerosis complex subunit 1 (TSC1) in dendritic cells leads to mTOR disinhibition and consequent ACC upregulation, thereby lowing acetyl-CoA levels and attenuating the epigenetic imprinting of MHC-1 by acetyl-CoA regulated histone acetylases [62]. Such changes in dendritic cells compromise their induction of activated CD8+ T cells and highlight the importance of acetyl-CoA to dendritic cell regulation of CD8+ T cells [62] and NK cells [63]. As with other immune cells, variations in glycolysis and OXPHOS are crucial to dendritic cell function, with consequences for the interactions of innate and adaptive immunity [64].

The AhR is expressed on dendritic cells where it significantly modulates dendritic cell function, indicating that AhR ligand upregulation will be an important modulator of all immune cells and their interactions in the tumour microenvironment [65]. This is also exemplified in MDSCs, where the AhR induces metabolic activation, via increased glycolysis and maintained OXPHOS, leading to heightened immune suppression [66]. This suggests that the release of kynurenine by tumours will have some co-ordinating effect on the metabolism and patterning of interactions among cells of the tumour microenvironment, with effects that may ultimately be associated with variations in the cytotoxic response of NK cells and CD8+ T cells. The concurrent regulation of acetyl-CoA and the melatonergic pathway within the cells of the tumour microenvironment clearly requires investigation.

### 3.2. Ageing, Acetyl-CoA, AhR and Sirtuins

SIRT1 regulates dendritic cell, NK cell and CD8+ T cell function, as well as the function of iNKT lymphocytes [67] and MDSC [68]. The loss of SIRT1 markedly upregulates ACC, decreasing acetyl-CoA and dramatically altering dendritic cell function [69]. As SIRT1, like melatonin, decreases over age, an ageing-associated decrease in SIRT1 will contribute to ACC elevations that lower acetyl-CoA, leading to a dysregulation in the co-ordination of glycolysis and OXPHOS [70]. Notably, the AhR increases with age and is proposed to be a major driver of ageing and ageing-associated medical conditions, including most cancers [71], as well as increases in ageing-associated fatality risk to SARS-CoV-2 infection and other age-linked medical conditions [1]. AhR activation also decreases SIRT1 in immune cells [72]. This is important to the regulation of mitochondria-located SIRT3 via the SIRT1 deacetylation and activation of SIRT3, and therefore mitochondrial function and SOD2 levels. Overall, ageing-associated changes in sirtuins, AhR, SOD2, and acetyl-CoA may be intimately linked to suboptimal cytolytic cell function, as an aspect of the changes underpinning immunosenescence, and contributing to alterations in the functioning and interactions of the cells of tumour microenvironment. Ageing-associated changes in sirtuins, acetyl-CoA, the melatonergic pathway and metabolism therefore modulate the function and interactions of the cells of the tumour microenvironment.

### 3.3. Circadian Dysregulation

Many factors dysregulate the circadian rhythm, including an increase in pro-inflammatory cytokines [73], amyloid-beta (Aβ) [74], gut-derived lipopolysaccharide (LPS) and high-mobility group box (HMGB1) [75], as well as circadian disruption associated with shift-work [76]. All of these factors are associated with decreased pineal gland melatonin production. Clinical and preclinical data indicate a role for circadian dysregulation in the aetiology and progression of tumours, with circadian disruption increasing the levels of CSC, proliferation and metastasis [77]. Variations in the circadian rhythm, including pineal melatonin, complicate the dynamic interactions of the cells in the tumour microenvironment [78]. Pineal melatonin upregulates Bmal1/PDC/acetyl-CoA/OXPHOS in immune cells, where under physiological conditions pineal melatonin may act to ‘reset’ the metabolism of immune cells. Consequently, factors acting to suppress pineal melatonin, such as pro-inflammatory cytokines, Aβ, LPS and HMGB1 will modulate the metabolism and interactions of the cells of the tumour microenvironment, adding another layer of complexity. The reciprocal negative interactions of melatonin and the AhR, coupled to the circadian rhythm of the AhR, would indicate that suppressed melatonin will significantly modulate the levels and effects of the AhR in the tumour microenvironment over the circadian rhythm. As such, suppressed pineal melatonin will modulate the AhR influence on the tumour microenvironment, including over the circadian rhythm.

Notably, pineal melatonin upregulates the alpha 7 nicotinic acetylcholine receptor (α7nAChR) levels. The α7nAChR significantly regulates immune cells and mitochondrial function [79], including from vagal ACh, allowing vagal ACh to afford protection against cancer progression via α7nAChR activation [80]. This would indicate that the suppression of pineal melatonin’s induction of the α7nAChR will contribute to alterations in the circadian regulation of the tumour microenvironment. Melatonin, the α7nAChR and the AhR are present on the mitochondria membrane [81], where their interactions will be important to determine. α7nAChR effects are complicated by its uniquely human duplicate, dupα7 (CHRFAM7A), which negatively regulates the α7nAChR. As the α7nAChR and dupα7 are differentially regulated, both genetically and epigenetically, this further complicates the interactions of melatonin, α7nAChR and dupα7 in the regulation of immune cells and mitochondrial function [82]. LPS can differentially regulate the α7nAChR and dupα7 [82], with LPS also inhibiting pineal melatonin, dysregulating immune responses and altering O-linked-N-acetylglucosaminylation (O-GlcNAcylation) [83]. O-GlcNAcylation is a significant regulator of tumours and cells of the tumour microenvironment. Such LPS effects would suggest that gut permeability-associated elevations in LPS may co-ordinate a number of important changes in the tumour microenvironment, including relative α7nAChR and dupα7 levels, with consequences for the circadian regulation of immune cells.

## 4. Wider Regulators of the Tumour Microenvironment and Immune Suppression

A number of other factors are frequently associated with the regulation of immune suppression in cancers, including yin yang (YY)1, protein phosphatase (PP)2A, glycogen synthase kinase (GSK)3β, ribosomal receptor for activated C-kinase 1 (RACK1) and the NOD-, LRR- and pyrin domain-containing protein 3 (NLRP3) inflammasome. Many of these factors have their pro-cancer effects significantly strengthened by O-GlcNAcylation, concurrent to alterations in acetyl-CoA and the melatonergic pathway.

### 4.1. O-linked-N-Acetylglucosaminylation (O-GlcNAcylation)

O-GlcNAcylation is a form of glycosylation, whereby a monosaccharide, viz O-GlcNAc, is added to a serine or threonine residue of nuclear and/or cytoplasmic protein by O-GlcNAc transferase (OGT). This is readily reversibly by the removal of O-GlcNAc by O-GlcNAcase (OGA). O-GlcNAcylation acts to regulate a wide array of physiological and pathophysiological processes by virtue of its ability to interact with nutrient sensing and metabolism, including via a host of signal transduction and transcription factors.

O-GlcNAcylation and the voltage-dependent anion channel (VDAC)2 are linked to the regulation of the apoptotic threshold during different physiological processes, including on mitochondria during the transfer of astrocyte mitochondria [84] and mitosis [85]. The O-GlcNAcylation of VDAC2 regulates the association of VDAC2 with Bcl-2-like family proteins, such as Bax and Bak, thereby modulating apoptotic susceptibility [86,87]. The O-GlcNAcylation of VDAC2 is therefore a significant determinant of mitochondria survival and presumably function during major physiological processes [88], including tumour survival via the regulation of chemotherapy resistance [86].

O-GlcNAcylation enhances the development, as well as the activation and proliferation of neutrophils, T and B cells, with regulatory effects in macrophages, whilst inhibiting the development and cytotoxicity of NK cells [89]. O-GlcNAcylation significantly regulates many of the transcriptional and translational processes that underpin fast effector CD8+ T cell proliferation, as well as a more limited number of studied proteins in memory-like CD8+T cells [90]. As such, variations in O-GlcNAcylation regulate the function of many cells in the tumour microenvironment, with some distinct and opposing effects in NK cells and CD8+ T cells. The relevance of O-GlcNAc to the regulation and dynamic interaction of cytolytic cells within the tumour microenvironment requires further investigation.

### 4.2. O-GlcNAcylation and Yin Yang1

The YY1 transcription factor significantly increases tumour cell growth and metastasis [91]. YY1 also induces immunosuppression, including by suppressing IFNγ [92], as well as upregulating PD-1, Lymphocyte-activation gene (Lag)3, and T cell immunoglobulin and mucin-containing domain (TIM)-3, indicating a significant role for YY1 in co-ordinating many of the correlates of ‘exhaustion’ [93]. O-GlcNAcylation increases YY1 stability, thereby stimulating YY1-dependent transcriptional activity, including of AANAT and the melatonergic pathway [94,95,96]. This would indicate that O-GlcNAcylated YY1 will interact with AhR activation to increase the NAS/melatonin ratio, and thereby NAS trophic support for tumours [8]. As YY1 upregulates TGF-β induced proliferation and migration in breast cancer cells [97], and O-GlcNAcylation stabilises TGF-β pathways [98,99], O-GlcNAcylation will have multiple effects on factors that promote tumour proliferation and immune suppression.

By inhibiting Bmal1 [100,101], YY1 will also contribute to how circadian dysregulation induces immune suppression, in association with an increase in PD-1 in cytolytic cells [102]. As pineal melatonin requires Bmal1 to drive the conversion of pyruvate to acetyl-CoA by PDC, YY1 and its potentiation by O-GlcNAcylation will modulate mitochondrial metabolism and its interactions, via acetyl-CoA, with glycolysis, thereby increasing key factors in these cells that regulate immune suppression and cytolytic cell ‘exhaustion’. YY1, like AhR activation, will therefore inhibit pineal melatonin’s ‘resetting’ of immune cell metabolism over the circadian rhythm. YY1 can also promote cytolytic cell ‘exhaustion’ via COX2 [103], PD-L1 induction [104] and the epithelial-mesenchymal transition (EMT) [105], whilst adenosine effects include YY1 induction [106]. This would indicate a positive feedback loop, whereby the YY1 upregulation of TGF-β effects, including adenosine induction in MDSCs will lead to adenosine induction of YY1, with this feedback loop being positively regulated by O-GlcNAcylation.

As well as increasing TGF-β in cancer cells [105], YY1 also increases TrkB levels in subsets of cancer cells, as shown in the squamous cell carcinoma (SCC)-25 cell line [107]. As noted, TrkB levels are increased in breast and glioma CSC, where TrkB activation contributes to CSC survival, proliferation and treatment resistance [18]. This is another CSC survival cycle that is increased by the O-GlcNAcylation of YY1, which concurrently enhances immunosuppression via TGF-β1 effects, including via the induction of MDSC adenosine leading to A2Ar activation-driven ‘exhaustion’ in NK cells and CD8+ T cells [108]. O-GlcNAcylation and YY1 may therefore be important co-ordinators of tumour progression and immune suppression, driven by alterations in metabolism and the melatonergic pathway in cells of the tumour microenvironment.

### 4.3. O-GlcNAcylation: PFK1, HIF-1α, PGK1 and Sirtuins

Hypoxia is proposed to afford a growth advantage to tumours partly mediated by the O-GlcNAc potentiation of the glycolytic enzyme, phosphofructokinase 1 (PFK1) at S529, thereby driving the pentose phosphate pathway in the generation of NADPH and GSH, which protects tumours from raised ROS levels [109]. O-GlcNAcylation also protects HIF-1α from proteasomal degradation, thereby enhancing tumour aerobic glycolysis [110] and protecting tumours against apoptosis [111], including from decreased epigenetic regulation by HDAC1 [112],

### 4.4. O-GlcNAcylation: Sirtuins and Metabolism

As noted, SIRT1 regulates immune cells [67,68], as well as ageing-associated changes in acetyl-CoA and OXPHOS [70]. SIRT1 also acts via the deacetylation and activation of mitochondria-located SIRT3. SIRT3 enhances mitochondrial respiration and lowers ROS production, whilst upregulating acetyl-CoA, and therefore mitochondrial OXPHOS, TCA cycle and melatonergic pathway [21]. SIRT3 directly interacts with PDC to increase its enzymatic activity, and therefore potentiates the conversion of pyruvate to acetyl-CoA [113], whilst also increasing PDC via Bmal1 upregulation [114], and thereby optimising the circadian regulation of metabolism. Consequently, SIRT1 and SIRT3 are important regulators of tumour cell survival [115,116] and cell function in the tumour microenvironment [117,118], with SIRT1 overexpression in mesenchymal stem cells (MSC) enhancing NK cell attraction, thereby shifting MSC effects from potentiating to supressing tumour progression [119]. SIRT1 effects are cell-dependent, allowing SIRT1 and SIRT3 to have differential effects in the cells of the tumour microenvironment [117]. O-GlcNAcylation of SIRT1 lowers SIRT1 levels and activity in an AMPK-dependent manner, with O-GlcNAcylated SIRT1 inhibiting the proteasomal degradation of oncogenic transcription factors [120] and SIRT3 activity, thereby lowering SIRT1- and SIRT3-driven PDC disinhibition, OXPHOS, TCA cycle, ATP and acetyl-CoA availability as well as lowering SOD2 and melatonergic pathway activation.

Whether the O-GlcNAcylation of SIRT1 is co-ordinated with the O-GlcNAcylation of YY1 and VDAC2, thereby enhancing YY1 transcription and VDAC2 protection against mitochondrial apoptosis, requires investigation. Clearly, any potentiation of YY1-induced AANAT, if coupled to AhR-induced CYP1B1 and the NAS activation of the BDNF receptor, TrkB, could allow co-ordinated O-GlcNAcylation to provide trophic support to tumours, whilst protecting mitochondria, limiting OXPHOS, maintaining HIF-1α and upregulating PFK1, PGK1 [121] and therefore glycolysis and the pentose phosphate pathway [110]. As c-MYC, including via the PI3K/Akt/mTOR/c-MYC pathway [122], is sufficient to upregulate O-GlcNAc and OGT in tumours, whilst mTORC1-induced c-MYC and its induction of LAT-1 are core aspects of glycolytic metabolism, changes driven by O-GlcNAcylation are integral aspects of the alterations in metabolism classically associated with tumours, namely glycolysis upregulation and the limited maintenance of OXPHOS. The differential effects of O-GlcNAcylation in tumours and NK cells may be a crucial aspect to the development of immune suppression amongst the cells of the tumour microenvironment. Shifts in these processes, coupled to sirtuin regulation, would not only underpin the tumour phenotype but would then alter the metabolism-driven tumour fluxes that influence metabolic responses in the cells of the tumour microenvironment. O-GlcNAcylation is therefore important to dynamic, intercellular aspects of metabolism, including via, but not limited to, sirtuin regulation.

### 4.5. O-GlcNAcylation: Protein Phosphatase (PP)2A, AhR, and mTORC1

Protein phosphatases (PP), especially PP2A, are long recognised as important regulators of the tumour microenvironment [123], including cytolytic cells, with PP2A inhibiting granzyme B and cytotoxicity in NK cells [124]. PP2A is also a powerful regulator of the AhR [125], with PP2A and mTORC1 being in a negative reciprocal interaction [126], indicating that PP2A will inhibit mTORC1-induced LAT1 and glycolysis, thereby driving ‘exhaustion’ in NK cells.

PP2A dephosphorylates and therefore activates ACC, thereby decreasing acetyl-CoA levels [127], indicating that PP2A may modulate the interactions of mitochondrial OXPHOS with the acetyl-CoA pathway driving glycolysis upregulation, as well as decreasing acetyl-CoA for the melatonergic pathway. As such, the negative reciprocal interactions of mTORC1 and PP2A in glycolytic cells may involve a PP2A-induced decrease in the acetylation of Raptor, thereby leading to the mislocalisation of mTORC1, a key aspect of ‘exhaustion’ in NK cells and CD8+ T cells. The interactions of miR-375 with PP2A may be important, as miR-375 disinhibits PP2A suppression from Cancerous Inhibitor of PP2A (CIP2A) [128], whilst miR-375 also suppresses 14-3-3ζ, and therefore prevents 14-3-3ζ from stabilising AANAT and initiating the melatonergic pathway [22,129]. Overall, PP2A decreases acetyl-CoA, the melatonergic pathway and the acetylated Raptor/mTORC1/glycolysis pathway, with PP2A levels and effects modulated by miR-375, in co-ordination with suppression of 14-3-3ζ and the melatonergic pathway.

PP2A can be O-GlcNAcylated, thereby regulating its function [130]. PPP2R2A is a major regulatory subunit of PP2A, with PPP2R2A promoting cancer cell survival and proliferation. The downregulation of PPP2R2A results in an elevation in the total levels of cellular O-GlcNAcylation, as shown in breast cancer cells [131]. This would suggest that any protection that the above would indicate as arising from decreasing PP2A, would be offset by the consequences of wider O-GlcNAcylation. This would indicate an array of intra- and intercellular options for the maintenance of immune suppression in the tumour microenvironment.

### 4.6. O-GlcNAcylation: RACK1 and NLRP3 Inflammasome

Ribosomal receptor for activated C-kinase 1 (RACK1) is associated with chemoresistance and tumour growth [132], with increased levels of RACK1 correlating with tumour progression and fatality [133]. RACK1 may act via a variety of processes in tumours, including upregulating the assembly and activity of the NLRP3 inflammasome [132,134], miRNAs patterning [135], and AhR regulation [136]. Being a ribosomal protein, RACK1 modulates how ribosomes spatiotemporally coordinate patterned gene expression, including local translation process [137] and centrosome regulation [138,139]. RACK1 stabilises PP2A [140], and PP2A-associated immune suppression [123]. RACK1 also induces immune suppression by increasing the M2/M1 macrophage ratio [141] and suppressing the numbers of CD4+, CD8+, and iNK T cells [142], as well as promoting the EMT [143]. As RACK1, in interaction with GSK3α, can regulate the circadian clock in mammalian cells [144], alterations in RACK1 levels and its interactions are likely to have wider circadian-driven immune-regulatory consequences. RACK1 therefore regulates many tumour associated pathways [145,146].

The O-GlcNAcylation of RACK1 increases its stability [147] and effects [132,148], including its association with PKCβ2, thereby enhancing eukaryotic translation initiation factor 4E phosphorylation and the translation of numerous, potent oncogenes [132]. RACK1 is a component of the NLRP3 inflammasome, increasing IL-1β and IL-18 levels [132,134]. Elevations in O-GlcNAcylation are associated with increased NLRP3 inflammasome and NF-κB signalling activity [147], with the NLRP3 inflammasome important to cancer progression and tumour microenvironment regulation [132], with effects at least partly regulated by the O-GlcNAcylation of RACK1.

RACK1 may also be regulated by YY1 [149], indicating wider interactions with factors known to regulate the tumour microenvironment, including the melatonergic pathway. The RACK1 upregulation of the NLRP3 inflammasome, IL-1β and IL-18 will induce the IDO/kynurenine/AhR pathway and therefore melatonergic pathway suppression. The O-GlcNAcylation of RACK1 may therefore modulate the melatonergic pathway in the tumour microenvironment via both YY1 and NLRP3. Although it is the effects of O-GlcNAcylated RACK1 on the NLRP3 inflammasome that are thought to underlie its regulation of tumours, tumour microenvironment and immune therapy response [150], the interactions of O-GlcNAcylated RACK1 on circadian, YY1, PP2A, acetyl-CoA and the melatonergic pathway may better integrate the effects of RACK1 with other tumour regulatory factors and processes, including metabolic.

### 4.7. O-GlcNAcylation: Mesenchymal Stem Cells (MSC)

MSC can upregulate tumour PD-L1 [151] and are important tumour microenvironment regulators [152]. MSC can self-renew, have a multidirectional differentiation potential, and efflux large quantities of exosomes, thereby potentially acting as a hub to integrate and influence signals across the tumour microenvironment [153]. MSC can release mitochondria, both directly and within extracellular vesicles, which can be taken up by other cells, which may be a treatment target [154]. MSC can also regulate the mitochondrial function of other cells [155]. MSC are regulated by circadian genes [156] and melatonin, with melatonin regulating OXPHOS [157], SIRT1/SOD2 [158] and exosome/vesicle content [159,160]. This would indicate that the circadian regulation of mitochondria by melatonin/SIRT1/SIRT3/Bmal1/PDC/OXPHOS pathway will modulate the phenotype of transferred mitochondria, with consequences for the functioning of cells uptaking such potentially distinct mitochondria ‘phenotypes’. It requires clarification as to whether increased VDAC2, and its O-GlcNAcylation, afford protection in the process of mitochondria transfer. The relevance of variations in MSC exosomes and mitochondria ‘phenotypes’ in tumours and other cells of the tumour microenvironment will be important to determine, including over the circadian rhythm [159], given the known immune regulatory effects of MSC exosomes [161].

Preclinical in vivo data on mitochondria transfer into rodent tumours show such transfer to significantly alter the tumour microenvironment, including by lowering oxidative stress, tumour size and enhancing immune cell infiltration [162]. Such data clearly indicate the importance of targeting metabolic processes in changing the tumour microenvironment. It will be important to clarify the impact of different mitochondria ‘phenotypes’, including as arising from alterations in the location and presence of the melatonergic pathway.

MSC are also important inducers of IDO, which is stably upregulated by the O-GlcNAcylation of signal transducer and activator of transcription 1 (STAT1) in these cells [163]. As IDO-induced kynurenine inhibits NK cells and CD8+ T cells via AhR activation [12], this is another route whereby O-GlcNAcylation in MSC, as well as in CSC [12], may modulate immune suppression in the tumour microenvironment. Interestingly, heightened O-GlcNAc in MSC is coupled to increased glycolysis [163], indicating that O-GlcNAcylation in MSC will modulate the interface between OXPHOS and glycolysis in these cells. The roles of alterations in acetyl-CoA and the melatonergic pathway in determining the MSC influence on the tumour microenvironment as well as exosomal content and mitochondria ‘phenotypes’ will be important to determine.

### 4.8. O-GlcNAcylation: Glycogen Synthase Kinase (GSK)3β and Cytolytic Cells

GSK3β inhibitors increase the cytotoxicity and maturation of NK cells and CD8+ T cells [164,165], coupled to PD-1 suppression [166]. GSK3β inhibition underpins the co-stimulatory effects of CD28 on CD8+ T cell cytotoxicity [167], whilst ligand activation of the NK cell activator receptor, NKG2D, is prevented by GSK3β [168]. CD28 co-stimulatory effects on CD8+ T cells increase mitochondria fusion, membrane potential and mitochondrial mass [169], highlighting the impact of GSK3β inhibition on mitochondrial metabolism in driving cytotoxicity, which is also evident from the increased acetyl-CoA and TCA cycle activity, as well as glycolysis, required for the initial activation of memory CD8+ T cells [170].

In contrast to NK cells, heightened O-GlcNAcylation enhances the functional response of CD8+ T cells [89,90], which may be mediated via the inhibitory effects of O-GlcNAcylation on GSK3β function [171]. As GSK3β inhibition also increased NK cells cytotoxicity, this would indicate that the differential effects of O-GlcNAcylation in NK cells and CD8+ T cells are not mediated via the O-GlcNAcylation, and inhibition, of GSK3β [172,173].

GSK3β is also intimately linked to the melatonergic pathway, with GSK3β inhibition attenuating IFNγ induction of IDO, as shown in dendritic cells [174], thereby decreasing kynurenine production for AhR activation and increasing serotonin availability for the melatonergic pathway. The O-GlcNAcylation of GSK3β may enhance more optimal pathway activity in cytolytic cells, including the melatonergic pathway. GSK3β inhibition prevents IFNγ-induced IDO [174], including in cancer cells [175], and dendritic cells [176]. Such inhibition involves suppression of the Janus-activated kinase (JAK)1/PKCδ/STAT1 pathway, which is also inhibited by green tea’s epigallocatechin gallate (EGCG) [175] and curcumin [176], as well as other GSK3β inhibitors [174]. GSK3β inhibition, including by O-GlcNAcylation, will therefore modulate both cancer cells and cytolytic cells.

**Figure 2:**
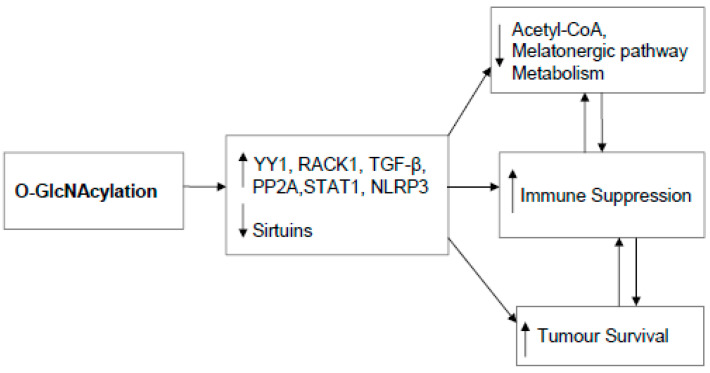
Shows how O-GlcNAcylation increases the activity of factors driving tumour survival and immune suppression of NK cells and CD8+ T cells, including by enhancing the activity of YY1, RACK1, TGF-β, PP2A, STAT1 and NLRP3, whilst inhibiting the beneficial effects of the sirtuins. All of these factors are also associated with the regulation of the melatonergic pathway, highlighting the importance of this pathway in driving the pathophysiological changes underpinning tumour survival and proliferation as well as immune suppression in cytolytic cells. Whether these factors, such as YY1 and RACK1, show co-ordinated O-GlcNAcylation in tumours and/or cytolytic cells awaits further investigation. It should also be noted that O-GlcNAcylation can inhibit GSK3β, thereby decreasing tumour survival and enhancing cytotoxicity in CD8+ T cells, indicating some contrasting effects of O-GlcNAcylation in NK cells. Abbreviations: Acetyl-CoA: acetyl-CoEnzyme A; NLRP3: NOD-, LRR- and pyrin domain-containing protein 3; O-GlcNAcylation: O-linked-N-acetylglucosaminylation; PP2A: protein phosphatase 2A; RACK1: ribosomal receptor for activated C-kinase 1; STAT: signal transducer and activator of transcription; TGF: transforming growth factor; YY1: yin yang 1.

GSK3β inhibition has reciprocal positive interactions with melatonin, with melatonin increasing the PI3K/Akt and ser9 phosphorylation and inhibition of GSK3β and GSK3β inhibition suppressing IDO [174]. However, a STAT1 binding site is present in the AANAT promotor [177], allowing STAT1 activation of IDO by IFNγ to induce the melatonergic pathway and melatonin release. It is likely that the concurrent release of melatonin would negatively feedback on the IFNγ/GSK3β/JAK1/PKCδ/STAT1/IDO, although AhR/CYP1B1 via the backward conversion of melatonin to NAS would have contrasting effects via TrkB activation. Clearly, a number of factors and interacting processes have evolved to regulate mitochondria metabolism and the melatonergic pathway, arising from their significant influence on cellular and intercellular functions, including many of the key processes classically associated with CSC proliferation and immunosuppression in the tumour microenvironment. Figure 2 shows O-GlcNAcylation of tumour environment factors.

## 5. Integrative Model

The above collection of diverse data indicates the important, if not over-riding, role that variations in metabolism, including from acetyl-CoA and the melatonergic pathway, can have on cellular function and intercellular interactions within the tumour microenvironment. The dynamic complexity of the tumour microenvironment, and the specific variations occurring in particular tissues and organs [178] may then be seen as a dynamic flux of interactions that act to modulate metabolism, driven in part, if not primarily, by variations in acetyl-CoA and the melatonergic pathway. The importance of metabolism is indicated by the dramatic changes occurring in tumours and the tumour microenvironment arising from mitochondria transfer in preclinical models [162]. Although many aspects of mitochondrial function may be relevant, the current article has highlighted the powerful role for acetyl-CoA regulation, including in co-ordinating OXPHOS and glycolysis in NK cells and cytolytic cells, as well as the impact that acetyl-CoA has on the melatonergic pathway. Such an integrative model provides a ‘higher-order’ structure, comprised of a metabolic matrix dynamically communicating across the different cells of the tumour microenvironment, including tumours.

It is proposed that metabolic alterations drive the patterning of miRNAs that underpin and co-ordinate the changing fluxes and plasma membrane receptors expressed in individual cells, as exemplified by the miR-138 inhibition of PD-1. Intercellularly, this metabolic power play is strongly driven by how various fluxes in the tumour microenvironment can influence acetyl-CoA and the melatonergic pathway in other cells. It would also suggest that metabolic interventions targeted to any cell in the tumour microenvironment would shift the pattern of interactions of all cells. This would seem more obvious in some cells, such as cancer cells or NK cells, than others, such as macrophages. Many of the factors significantly associated with tumour progression and immunosuppression, such as YY1, SIRT1/3, GSK3β, STAT, IDO and the AhR can have their influence and interactions more plausibly integrated by their impacts on metabolism, acetyl-CoA and the melatonergic pathway within the cells of the tumour microenvironment.

Clearly, factors acting to regulate the interactions of OXPHOS and glycolysis via acetyl-CoA and the melatonergic pathway may be variably important across the different cells of the tumour microenvironment. Although, requiring experimental investigation, the above would indicate that targeting metabolic processes, acetyl-CoA regulation and melatonergic pathway variations in NK cells may be the most viable treatment target for changing the intercellular interactions within the immune suppressed tumour microenvironment. NK cells are one of the early cells to interact with tumours and alterations in NK function seem important to tumour microenvironment development. This may arise as a consequence of how CSCs react to NK cell IFNγ efflux [Liu et al., 2018], especially to IFNγ-induced IDO and kynurenine, which upon release activates the AhR on NK cells and other cells in the emerging tumour microenvironment, thereby initiating a pattern of CSC influence on acetyl-CoA, the melatonergic pathway and metabolism in other cells.

The significant effects of O-GlcNAcylation in the regulation of CSC levels, survival and proliferation [179] are intimately linked to its regulation of the processes underlying variations in metabolic regulation and the melatonergic pathway, both within tumours and other cells of the tumour microenvironment. TrkB+CSC are important to the survival, proliferation and recurrence of breast cancers and GBM [18,180], indicating that BDNF and any released NAS are crucial regulators of CSCs. The O-GlcNAcylation of YY1 and the upregulation of NAS seem important drivers of TrkB effects. Importantly, TrkB activation triggers the PI3K/Akt/mTOR and cMYC pathways that upregulate O-GlcNAcylation [181,182]. This is why the initial response in CSCs in regard to increasing kynurenine release and AhR activation is important, as any O-GlcNAcylation of YY1 and melatonergic pathway activation will have its effects dramatically determined by variations in the NAS/melatonin ratio. Consequently, biasing the melatonergic pathway to increase NAS or the NAS/melatonin ratio may result in heightened NAS activation of TrkB, leading to an increase in O-GlcNAcylation of crucial factors acting to regulate CSC and the wider tumour microenvironment. This would indicate a maintained positive feedback loop involving O-GlcNAcylation and factors increasing the NAS/melatonin ratio, especially AhR-induced CYP1B1, but also P2Y1r and mGluR5 activators, as well as regulators of O-demethylation, which all ‘backward’ convert melatonin to NAS. These processes clearly require further investigation, including as to whether cytotoxic cells can be converted to Trojan horses carrying and releasing trophic NAS for CSC maintenance and proliferation. How such processes act to upregulate BDNF will also be important to determine, including NAS-induced BDNF, as shown in the brain [18].

Such a perspective incorporates a lot of previously disparate data on tumours and the tumour microenvironment, some of which have been highlighted in this article. A number of treatment and future research implications arise from this.

## 6. Future Research

(1) Does tumour-derived kynurenine activation of the AhR on tumour microenvironment cells differentially regulate acetyl-CoA and the melatonergic pathway in these cells, including an increase in the NAS/melatonin ratio? Is NAS released and/or does NAS induce BDNF? Does this contrast to a total suppression of melatonin and the melatonergic pathway in CSCs?
(2) As melatonin release is important to the switching of macrophages and other immune cells to an anti-inflammatory M2-like phenotype [183], is the cytoplasmic induction of AANAT and ASMT relevant to modulating the activation state of CD8+ T cells and NK cells? Would factors, such as AhR-induced CYP1B1, increase NAS, rather than melatonin, release under such conditions, thereby affording protection of CSC? Is NAS always released with melatonin from immune cells, as occurs nightly in the pineal gland, such that variations in immune, and other cells, NAS/melatonin ratio may contribute to tumour initiation?
(3) As GSK3β inhibitors, including lithium, have long been known to increase NK cell and CD8+ T cells cytotoxicity [165], the interaction of GSK3β with the AhR, acetyl-CoA and the melatonergic pathway in NK cells and CD8+ T cells requires investigation.
(4) Caffeine elevates CD8+ T cell cytotoxicity, including by decreasing PD-1 [184]. Is this mediated by A2Ar inhibition and/or via metabolic impacts driving an increase in miR-138?
(5) Is there a circadian rhythm to the levels and/or content of MSC exosomes, including transferred mitochondria and mitochondria ‘phenotype’ via the melatonin/SIRT1/SIRT3/Bmal1/PDC/OXPHOS pathway?
(6) How important is pineal melatonin-induced sirtuins to the resetting of mitochondrial and cellular function in CD8+ T cells and NK cells over the circadian rhythm, and does the suppression of pineal melatonin over age contribute to ageing-associated cancer risk/severity?
(7) Is the O-GlcNAcylation of STAT1 and raised IDO and glycolysis levels co-ordinated with O-GlcNAcylation of VDAC2 and YY1 in CSC and MSC [163]? Does the GlcNAcylation of VDAC2 constitute a protected mitochondria phenotype, including of NK cells and other tumour microenvironment cells within such a challenging microenvironment?
(8) Is the AhR expressed on chimeric antigen receptor (CAR)-NK cells and does its activation/antagonism modulate the utility of CAR-NK cells in the treatment of an array of different cancers [3].
(9) Are there distinct ‘suppressed’ TIM-3+ PD-1+ Lag3+ CD8+T cells, versus ‘exhausted’ CD8+ T cells, as recently proposed [91]. Given that YY1 increases TIM-3, PD-1 and Lag3 [93], are ‘suppressed’ CD8+ T cells driven by YY1 and its O-GlcNAcylation, with ‘exhaustion’ driven by wider O-GlcNAcylated factors and metabolic regulators?
(10) Experimental circadian rhythm disruption increases tumour growth in association with alterations in the immune cells of the tumour microenvironment [185]. How important is the O-GlcNAcylated YY1 suppression of Bmal1 to the function and circadian rhythm of tumour microenvironment cells?
(11) How important are the gut microbiome-derived short-chain fatty acids, especially butyrate and acetate, in the regulation of metabolism, acetyl-CoA and the melatonergic pathway in the cells of the tumour microenvironment, as indicated by previous data [186,187].
(12) Is the differential regulation of the α7nAChR and dupα7 by LPS co-ordinated with alterations in the levels of O-GlcNAc, as some data could suggest [83]. The consequences of the differential expression of α7nAChR and dupα7 on immune activity, including the tumour microenvironment over the circadian rhythm, clearly require investigation, including as to any interactions with gut permeability-derived LPS.
(13) PD-1 is upregulated by many transcription factors and pathways [188], as well as kynurenine’s activation of the AhR [12]. All can be repressed by melatonin, whilst melatonin also upregulates many of the PD-1 repressors [188]. As to how these PD-1 inducing and repressing factors interact with acetyl-CoA, the melatonergic pathway and OXPHOS in CD8+ T cells and NK cells will be interesting to determine. For example, would the suppression of melatonin production within immune cells, perhaps via lost autocrine effects [183], prevent its beneficial effects on such immune checkpoint regulation?
(14) As a number of miRNAs, including miR-28, miR-138, miR-4717, and miR-374b, suppress PD-1 expression levels via direct binding to the 3′ UTR of PD-1 mRNA, the regulation of miRNAs is one of the ways by which alterations in metabolism may modulate PD-1 expression levels. Notably, miR-28, miR-138 and miR-347b [189,190,191] can also regulate glycolysis and metabolism. Clearly, the interface of miRNAs with metabolism, acetyl-CoA and the melatonergic pathway require investigation. The importance of this is highlighted by data showing miR-138 can also suppresses COX2 and PDK1, indicating that it will disinhibit the PDC, and increase the conversion of pyruvate to acetyl-CoA, whilst preventing PD-1 and COX2/PGE2/EP4-driven ‘exhaustion’ [114,192]. Clearly, miRNA patterning will be an important mediator of co-ordinated transcriptional responses to altered metabolic regulation, as indicated in Figure 1.
(15) Although taurine has a number of anticancer and anti-inflammatory effects [193], its induction of the lncRNA, taurine upregulated (TUG)1, leads to increased tumour proliferation, metastasis and survival [194]. TUG-1 also induces ACC, thereby lowering acetyl-CoA and inhibits miR-138 [195], and therefore the miR-138 inhibition of PD-1. TUG-1 also binds to the PGC-1α promotor, inhibiting PGC-1α induction of optimal mitochondrial function [196], indicating that it will have significant effects on metabolism and miRNA patterning. TUG-1 effects on acetyl-CoA, the melatonergic pathway, metabolism and miRNA patterning clearly require further investigation in cytolytic cells.
(16) Does racial discrimination stress have biological consequences relevant to the modulation of cancer risk and severity, including by acting on the tumour microenvironment, as recently proposed for similar processes in COVID-19 associated infection [1]? African-Americans, vs. European Americans, have an increased cancer risk, severity and fatality [197], which is typically explained by ethnic genetic diversity, decreased vitamin D, low therapy maintenance or lower maintenance of health insurance policies [198]. Heightened cytokine levels in African-Americans correlate with racial discrimination stress experiences, indicating the association of racism with increased kynurenine activation of the AhR, thereby priming for immune suppression [1]. This will be important to investigate and may indicate wider biological consequences of social stratification processes across different cultures.
(17) Some of the consequences of racial discrimination stress can overlap with PTSD [199]. PTSD is associated with a decrease in the CpG demethylation of the AhR repressor (AHRR) [200], indicating PTSD to interact with the epigenetic regulation of the AhR by the AHRR. PTSD also associates with increased cancer risk [201]. Given the overlaps of racial discrimination stress with PTSD, it requires investigation as to whether some of the impacts of racism on the crucial role of the AhR in cancers may be modulated by discrimination stress impacts on the CpG methylation status of the AHRR, and thereby on AhR regulation of tumours and the tumour microenvironment.
(18) Ten-eleven translocation (TET) are important drivers of cancer initiation and progression via the regulation of epigenetic processes [202]. O-GlcNAcylation potentiates TET effects, including via DNA demethylation and histone methylation [202]. The interactions of O-GlcNAcylated TET with the AhR/AHRR and mitochondrial function in cells of the tumour microenvironment will be important to determine, especially as this has been proposed to underpin AhR-driven active DNA demethylation and thereby epigenetic memory [203]. Notably, YY1 is a significant regulator of TET2 [204], and TET2 is an important aspect of chronic stress and its treatment [205], indicating that this could have relevance to how PTSD and racism modulate the AhR/AHRR and its possible association with racial health disparities. This is supported by preclinical data showing social stress to significantly increase TET [206].
(19) Both acute and chronic stress can exacerbate tumour initiation and progression [207,208]. Much of the research on stress has highlighted the role of alterations in sympathetic nervous system activity [208]. As to how alterations in the autonomic nervous system are co-ordinated with the cytokine/IDO/kynurenine/AhR pathway in the effects of acute and chronic stress, including racial discrimination stressors, will be important to determine.
(20) The AhR level and AhR-induced CYP1B1 levels are positively correlated with leptin in childhood obesity. Leptin primes cytolytic cells for attenuated cytotoxicity [7]. Whether the association of leptin, CYP1B1 and AhR ligands is correlated in adult obesity, in association with a decreased CD8+ T cell and NK cell cytotoxicity, as some data could suggest [209], will be important to determine.
(21) Work on T cell function indicates an important role for mitochondria calcium regulation [210], with mitochondrial calcium uptake (MICU)1 and MICU2, rather than mitochondrial metabolic activity per se, proposed as significant regulators of immune cell function [211]. Recent data show melatonin to prevent cellular pathology via MICU regulation [212], indicating that variations in pineal and local melatonin production will modulate the MICU influence on mitochondria-determined immune cell function. Given the role of MICU in tumour pathophysiology [213], the relevance of MICU regulation in the cells of the tumour microenvironment, including as regulated by melatonin, will be important to determine. Other aspects of mitochondria calcium regulation may be associated with melatonergic pathway regulation, given the Leucine zipper-EF-hand containing transmembrane protein 1 (LETM1) transporter on the inner mitochondria membrane has a 14-3-3-like motif that may bind and stabilise AANAT [214]. Consequently, the melatonergic pathway may be intimately linked to mitochondria calcium regulation. This could indicate that a mitochondrial calcium, rather than metabolic, regulation of the tumour microenvironment may be intimately linked to the regulation of the mitochondrial melatonergic pathway.
(22) Given that the SARS-CoV-2 virus may lead to changes in the immune responses that parallel those evident in the tumour microenvironment [1], it will be important to determine the influence of SARS-CoV-2 infection on tumour aetiology and progression.

## 7. Treatment Implications

Although cancer treatment has focussed primarily on killing cancer cells, including by inhibiting tumour mitochondrial function [215], it has long been realised that a more sophisticated treatment would be to change the nature of cellular interactions in the tumour microenvironment, thereby allowing NK cells, CD8+ T cells, CD4+ T cells and γδ T cells to eliminate tumours. The dynamic complexity of the intercellular interactions of the tumour microenvironment have thwarted such attempts. The past decade has seen a growing interest in the utilisation of immune checkpoint inhibitors, such as PD-1 inhibitors, in an attempt to achieve this, which have not fulfilled the expectations raised [216]. The integrative model above provides a framework that emphasises the importance of factors acting to regulate variations in cellular metabolism, especially acetyl-CoA and the melatonergic pathways, as major drivers of the ever-changing, dynamic fluxes occurring amongst cells in the tumour microenvironment. This is a conceptualisation that focusses on the complexity of the intracellular processes that act to regulate variations in metabolism, including the interactions of OXPHOS and glycolysis, whilst highlighting the important role of acetyl-CoA, the melatonergic pathway and the AhR. Such a conceptualisation indicates that current treatment targets, such as PD-1 inhibitors and adenosine A2Ar antagonists/antibodies, are treatments targeting factors that are downstream of metabolic-driven alterations in miRNA-driven gene expression patterning. This suggests that the fate of immune cells within the tumour microenvironment does not have to be determined by cancer cell-driven fluxes, which can be overcome by interventions targeting intracellular processes driving the complexity of metabolic regulation within immune cells.

Clearly, future research investigating fundamental aspects of metabolic processes, such as the role of increase NAS production and subsequent TrkB activation in driving CSC survival and proliferation, will help to provide more focussed treatment. In the absence of such data, a number of possible treatment implications may arise from the above.

(1) Although melatonin can kill most cancer cells, its utilisation has primarily been as an adjunctive, often to prevent side-effects of other medications. Treatments targeting the upregulation of local melatonin production across the cells in the tumour microenvironment, coupled to the suppression of factors such as CYP1B1 that backward convert melatonin to NAS, could allow melatonin to induce apoptotic processes in tumours as well as change the nature of the cellular interactions within the tumour microenvironment.
(2) It is seldom that treatments are utilised that take into account circadian factors. The re-instatement of a circadian rhythm within the cells of the tumour microenvironment, including the expression of Bmal1 and SIRT1, will modulate the cells of the tumour microenvironment, including by the utilisation of exogenous melatonin [217].
(3) Targeting MSC mitochondria release, both directly and within extracellular vesicles, for uptake in cells of the tumour microenvironment may be a viable treatment target [154].
(4) Given the importance of the immune response, it is clear that a more holistic conceptualisation of immune cell regulation may also guide more physiology-based treatments. The gut microbiome short-chain fatty acids levels are significant regulators of PD-1 inhibitor treatment of solid tumours [218]. This may be linked to the utility of butyrate as a HDAC inhibitor. However, butyrate also optimises mitochondrial function, by increasing PDC, acetyl-CoA and the melatonergic pathway [219,220]. This is supported by data showing butyrate supplementation to increase the efficacy of CD8+ T cells via metabolic impacts [187,188] as well as the cytotoxicity of NK cells against tumours [221]. As butyrate also inhibits YY1 [222], the adjunctive use of sodium butyrate would have clinical utility via a number of processes.
(5) The efficacy of a number of nutraceuticals in the beneficial regulation of the tumour microenvironment may also be placed within the above model. Nutraceutical antagonists of the AhR, including EGCG, resveratrol, folate, vitamin B12 and curcumin [1]. All have anticancer efficacy and modulate the interactions of the tumour microenvironment [223,224,225,226,227]. Notably, these factors can upregulate the melatonergic pathway [228]. EGCG and curcumin can also inhibit GSK3β [175,176]. The utilisation of multifunctional nanoparticles that target the tumour microenvironment will improve both efficacy and specificity of such effects [223].
(6) Quercetin may be another nutraceutical target. As quercetin suppresses ACC, thereby increasing acetyl-CoA, it may be mediating some of its antitumour benefits via a decrease in ‘exhaustion’ susceptibility, rather than an increase in basal or in vitro stimulated NK cell activation [229]. Quercetin and doxorubicin nanoparticles allow for increased efficacy, via the tumour microenvironment remodelling effects of Quercetin that allow greater doxorubicin penetration into tumour tissues [230].
(7) The utilisation of oncolytic viruses may help to turn ‘cold’ tumour microenvironments to ‘hot’ and therefore immune responsive [2]. Likewise, the elimination of solid tumour hypoxia by synthetic oxygenation agents and natural blood substitutes may block the hypoxia-HIF-1α-adenosine/A2Ar pathway, thereby enhancing immunotherapy-targeted treatments [44]. Preclinical in vivo data on mitochondria transfer into rodent tumours show such transfer to significantly alters the tumour microenvironment, including by lowering oxidative stress, tumour size and enhancing immune cell infiltration [162]. Such data clearly indicate the importance of targeting metabolic processes in changing the tumour microenvironment.

## 8. Conclusions

This article reviews the role of the mitochondrial and cytoplasmic melatonergic pathway in the tumour microenvironment and how this may be intimately linked to the wide array of factors and processes underpinning tumour suppression. The impacts of O-GlcNAcylation on tumour growth and tumour microenvironment cellular responses seem predominantly mediated by factors, such as YY1, and processes, such as OXPHOS and glycolytic metabolism, that are intimately linked to acetyl-CoA and the regulation of the melatonergic pathway. The capacity of CSCs to increase kynurenine release and AhR activation to the NK cell release of IFNγ may be important to the initiation and development of immune suppression in the tumour microenvironment, especially as NK cells are often the first cytolytic cell to encounter tumours. A pattern for the alterations in metabolism, as well as acetyl-CoA and melatonergic pathway regulation would then underpin and drive the dynamic intercellular fluxes of the tumour microenvironment.

It is also important to note that the tumour microenvironment is distinctly regulated in different organs and tissues, as highlighted by recent data on MDSC in different organs [178]. These authors showed cancer-associated MDSC cellular processes are significantly determined by organ-specific conditions [178]. Such data show the plasticity and complexity arising from the distinct and dynamic intercellular interactions occurring within the tumour microenvironment. Whether acetyl-CoA regulation and the melatonergic pathway within mitochondria form a crucial hub in determining such plasticity and complexity will be important to determine across different tumour microenvironments. This could suggest that treatments focussed on regulating acetyl-CoA and the mitochondrial melatonergic pathway may be targets, with the potential to over-ride the dynamic intercellular interactions within the tumour microenvironment across different organs and tissues.

## Abbreviations

α7nAChR	alpha 7 nicotinic acetylcholine receptor
Aβ	amyloid-beta
AANAT	aralkylamine N-acetyltransferase
ACC	acetyl-CoA carboxylase
Acetyl-CoA	acetyl-coenzyme A
AhR	aryl hydrocarbon receptor
AMPK	AMP-activated protein kinase
CD8+	cluster of differentiation 8
COVID-19	coronavirus disease-19
COX	cyclooxygenase
CRH	corticotropin-releasing hormone
CSC	cancer stem-like cells
CYP	cytochdrome P450
EGCG	epigallocatechin gallate
EP4	prostaglandin E2 receptor 4
FICZ	6-formylindolo[3,2-b]carbazole
HIF	hypoxia-inducible factor
HMGB	high-mobility group box
IDO	indoleamine 2,3-dioxygenase
IFN	interferon
IL	interleukin
JAK	Janus-activated kinase
LAG-3	lymphocyte-activation gene 3
LPS	lipopolysaccharide
MDSC	myeloid-derived suppressor cells
MHC	major histocompatibility complex
NAS	N-acetylserotonin
NK	natural killer
NLRP3	NOD-, LRR- and pyrin domain-containing protein 3
O-GlcNAc	O-linked-N-acetylglucosaminylation
OXPHOS	oxidative phosphorylation
PDC	pyruvate dyhydroganse complex
PD-1	programmed cell death-1
PD-L1	programmed cell death ligand 1
PDK	pyruvate dehydrogenase kinase
PGE2	prostaglandin E2
PP2A	protein phosphatase 2A
RACK1	ribosomal receptor for activated C-kinase 1
S1P	sphingosine-1-phosphate
SARS-CoV-2	severe acute respiratory syndrome coronavirus
SNP	single-nucleotide polymorphism
SOD	superoxide dismutase
STAT	signal transducer and activator of transcription
TCA	tricarboxylic acid
TCDD	2,3,7,8-tetrachlorodibenzo-p-dioxin
TDO	tryptophan 2,3-dioxygenase
TGF	transforming growth factor
TIM-3	T cell immunoglobulin and mucin domain 3
TLR	toll-like receptor
TNF	tumour necrosis factor
Treg	regulatory T cells
TUG	taurine upregulated
YY1	yin yang 1
